# p53 status modifies cytotoxic activity of lactoferrin under hypoxic conditions

**DOI:** 10.3389/fphar.2022.988335

**Published:** 2022-09-19

**Authors:** Maryami Yuliana Kosim, Takahiro Fukazawa, Mutsumi Miyauchi, Nobuyuki Hirohashi, Keiji Tanimoto

**Affiliations:** ^1^ Department of Radiation Disaster Medicine, Research Institute for Radiation Biology and Medicine, Hiroshima University, Hiroshima, Japan; ^2^ Natural Science Center for Basic Research and Development, Hiroshima University, Hiroshima, Japan; ^3^ Department of Oral and Maxillofacial Pathology, Graduate School of Biomedical and Health Sciences, Hiroshima University, Hiroshima , Japan

**Keywords:** lactoferrin, cytotoxic activity, hypoxia, p53, ferroptosis

## Abstract

Lactoferrin (LF) is an iron binding glycoprotein of the transferrin family with a wide spectrum of biological effects, including anti-cancer activity. However, the detailed molecular mechanisms of anti-cancer activity of LF have not been fully determined. In this study, we tried to clarify cytotoxic functions of LF on various cell lines under hypoxic conditions and elucidate those molecular mechanisms. Cytotoxic activity of LF on cell lines was found to have a range of sensitivities. Hypoxia decreased sensitivity to LF in KD (lip fibroblast) but increased that in HSC2 (oral squamous cell carcinoma). Expression analyses further revealed that LF treatments increased hypoxic HIF-1α, -2α and p53 proteins in KD but attenuated them in HSC2 cells, and decreased HIF-1 target gene, *DEC2*, in KD but increased it in HSC2, suggesting a possible relationship between LF-modified *DEC2* expression and HIF-α protein. MTT assay strikingly demonstrated that cells expressing mutant-type p53 (MT5) were more sensitive to LF than control HepG2 (hepatoma), suggesting an important role of the p53 signal. Knock-down of *TP53* (p53 gene) interestingly reduced sensitivity to LF in HepG2, suggesting that p53 may be a target of LF cytotoxic activity. Further analyses with a ferroptosis promoter or inhibitor demonstrated that LF increased *ACSL4* in hypoxic MT5, suggesting LF-induced ferroptosis in cells expressing mutant-type p53. In conclusion, hypoxia was found to regulate cytotoxic activities of LF differently among various cell lines, possibly through the p53 signaling pathway. LF further appeared to regulate ferroptosis through a modification of *ACSL4* expression.

## Introduction

Lactoferrin (LF) is an 80-kD iron binding glycoprotein of the transferrin family with a wide spectrum of biological effects, including anti-bacterial, anti-inflammatory, immunomodulatory, and anti-cancer activities (Rodrigues et al., 2009; Kruzel et al., 2017; Kell et al., 2020). LF is found in various fluids secreted from glandular systems, including tears, perspiration, and milk, so it is generally considered to be non-toxic (Iglesias-Figueroa et al., 2019). Bovine lactoferrin (bLF) is now approved as a nutritional product by the Therapeutic Goods Administration (TGA, Australia), the Food and Drug Administration (FDA, United States), and the European Food Safety Authority (EFSA). Purified bLF has been further proven in multiple mouse models to enhance production of interferon-γ (IFN-γ), caspase-1, and interleukin-18 (IL-18), as well as lead to an increase of immune cell numbers (Iigo et al., 2004; Takakura et al., 2006; Siqueiros-Cendón et al., 2014). bLF has been also reported to inhibit colon, esophagus, lung, and bladder carcinogenesis in rats when administered orally in the post-initiation stage, suggesting it as a potential therapeutic agent (Iigo et al., 1999; Ushida et al., 1999; Masuda et al., 2000; Tsuda et al., 2000; Tsuda et al., 2002; Ramirez-Rico et al., 2022). However, the detailed molecular mechanisms of anti-cancer activity of LF have not been fully determined.

Tissue hypoxia is well-known to contribute to biologically aggressive tumor phenotypes and emergence of therapeutic resistance through reprograming of glucose metabolism (Höckel and Vaupel, 2001; Muz et al., 2015; Petrova et al., 2018; Sørensen and Horsman, 2020). In solid tumors, cancer cells are often exposed to low oxygen tension (hypoxia), low pH, and low nutrition due to inadequate vasculature, which cause a variety of biological changes through alterations of the expression of various genes (Keith and Simon, 2007; Jing et al., 2019; Abou Khouzam et al., 2021). Hypoxia-inducible factor-1α and -2α (HIF-1α and HIF-2α) are key transcription factors regulating a variety of hypoxia-inducible genes. Activated HIFs interact with cognate hypoxia-response elements (HRE) of target gene promoters under hypoxic conditions. To date, many HIF-target genes have been identified and their functional significance has been analyzed in a variety of cancers (Semenza and Wang, 1992; Wincewicz et al., 2007; Kaluz et al., 2009; Liu et al., 2012; Albanese et al., 2021; Kunej, 2021). We have identified several novel hypoxia-inducible genes and revealed their functions related to response to radiation- and chemo-therapy (Miyazaki et al., 2002; Nakamura et al., 2008; Shirakura et al., 2010; Bhawal et al., 2011; Khalesi et al., 2013; Nakamura et al., 2018). Hypoxia is also known to induce stress in the tissue microenvironment, which activates p53 signals (Zhang C et al., 2021). Tumor suppressor p53 protein works as a guardian of genome stability, regulator of the cell cycle, and conveyor of cell death signals including apoptosis and ferroptosis (Liu and Gu, 2022). Ferroptosis is a recently identified mode of cell death that is initiated by oxidative perturbations of the intracellular microenvironment and can be inhibited by iron chelators and lipophilic antioxidant (Galluzzi et al., 2018). Because LF itself is a natural iron chelator, albeit a more hydrophilic antioxidant (Mulder et al., 2018; Bielecka et al., 2022), it is expected to modify ferroptosis, especially in cancer cells (Zhang Z et al., 2021).

In this study, we undertook to clarify the cytotoxic functions of LF on various cell lines under hypoxic conditions and further understanding of those molecular mechanisms. LF was found to have cytotoxic activities on hypoxic cells with a range of sensitivities, possibly involving the p53-signaling pathway.

## Materials and methods

### Chemicals

Most chemicals were analytical grade and were purchased from FUJIFILM Wako Pure Chemicals (Osaka, Japan), Sigma-Aldrich (St. Louis, MO, United States). Bovine lactoferrin (FUJIFILM Wako Pure Chemicals), Erastin (Selleckchem, Huston, TX, United States), or UAMC-3203 (Selleckchem). Bleomycin was kindly provided by Nippon Kayaku Company.

### Cell lines

Human cell lines TIG-3 (lung fibroblast), KD (lip fibroblast), HSC2 (oral squamous cell carcinoma), MCF-7 (breast cancer), HeLa (cervical cancer), and HepG2 (hepatoma) were purchased from American Type Culture Collection (ATCC) or the Japanese Cancer Research Resource Bank (JCRRB) and have been maintained as original stocks. The cells were normally maintained in MEM, DMEM, or RPMI1640 (NACALAI TESQUE, Inc., Kyoto, Japan) containing 10% fetal bovine serum (FBS; BioWhittaker, Verviers, Belgium). HepG2 cells expressing mutant-type p53 were generated as previously described (Fukazawa et al., 2019). Briefly, pCMX empty plasmid vector or pCMX-p53-R248W were stably transfected into HepG2 cells, and stable clones were selected with medium containing G418 (C4: pCMX transfected cells; MT5: p53-R248W transfected cells). For expression analyses, cells (1 × 10^6^ cells/10 cm diameter dish) were treated without or with the indicated concentrations of LF and subsequently cultured under normoxic (21% O_2_) or hypoxic (1% O_2_ in hypoxic chamber) conditions for 24 h. They were then harvested *via* centrifugation and cell pellets were store at −80°C until use.

### Cytotoxicity assay

Drug-induced cytotoxicity was evaluated with a conventional MTT dye reduction assay. Briefly, 4 × 10^3^ cells were seeded in each well of 96-Micro Well Plates (NUNCLON, NUNC, Roskilde, Denmark) with regular medium. After incubation without or with LF or UAMC-3203 for 24 h, cells were exposed to various concentrations of LF, Bleomycin, or Erastin, for 72 h under normoxic or hypoxic conditions. Then 10 μl of 0.4% MTT reagent and 0.1 M sodium succinate were added to each well, and after 2 h incubation, 150 μl of DMSO was added to dissolve the purple formazan precipitate. The formazan dye was measured spectrophotometrically (570–650 nm) with a MAXline^®^ Microplate Reader (Molecular Devices Corp., Sunnyvale, CA, United States). The cytotoxic effect of each treatment was assessed by IC_50_ value (inhibitory drug concentration producing 50% cell growth: drug concentration of 50% optical density of control). For knock-down experiments, nonspecific (siNS, No. 1027310), *DEC2* (siDEC2, SI00428008), or *TP53* (siTP53, SI0051779) siRNA (QIAGEN, Inc., Valencia, CA) was transfected with Lipofectamine^TM^ RNAiMAX (Thermo Fisher Scientific Inc.) into HepG2 cells (2 × 10^3^/well of 96-Micro Well Plates) for 4 h, and then the cells were treated without or with LF under normoxic or hypoxic conditions for 72 h. Cell viabilities were evaluated with the MTT assay as above, and measured spectrophotometrical values were compared.

### Immunoblot analysis

To analyze protein expression, whole cell extracts were prepared from cultured cells with or without hypoxic treatment as previously described (Tanimoto et al., 2000). 50 μg of extracts was blotted onto nitrocellulose filters following SDS-polyacrylamide gel electrophoresis. Anti-HIF-1α (610959, BD Pharmingen, San Diego, CA, United States), anti-HIF-2α (#7096, Cell Signaling TECHNOLOGY, Danvers, MA, United States), anti-E-cadherin (GTX100443, GeneTex, Irvine, CA, United States), anti-N-cadherin (GTX127345, GeneTex), anti-Vimentin (O91D3, BioLegend Inc., San Diego, CA, United States), anti-p53 (Ab-6, #OP43, ONCOGENE RESEARCH PRODUCTS, Boston, MA, United States), or anti-β-actin (A5441, Sigma) were used as primary antibodies, diluted 1:5000, 1:1000, 1:1000, or 1:5000, respectively. A 1:2000 dilution of anti-mouse IgG or anti-rabbit IgG horseradish peroxidase conjugate (#7076, #7074, Cell Signaling TECHNOLOGY) was used as a secondary antibody. Immunocomplexes were visualized by using the enhanced chemiluminescence reagent SuperSignal West Pico PLUS (Thermo Fisher Scientific K.K, Tokyo).

### RNA preparation and quantitative RT-PCR analysis

Total RNA was prepared from frozen cell pellets by using NucleoSpin^®^ RNA (MACHEREY-NAGEL GmbH&Co., KG, Düren, Germany) according to the manufacturer’s instructions. To avoid detecting genomic or plasmid DNA with RT-PCR, all of the RNA samples were treated with DNase, which was included in the kit. 1 µg of total RNA was reverse-transcribed using a High-Capacity cDNA Archive^TM^ Kit (Applied Biosystems, Foster City, CA, United States). A two-hundredth aliquot of the cDNA was subjected to quantitative RT-PCR using primers (final concentration 200 nM each) and MGB probe (final concentration 100 nM; the Universal Probe Library [UPL], Roche Diagnostics, Tokyo, Japan) sets (Supplementary Table S1) for *CA9*, *DEC1*, *DEC2*, *BCL2*, *BAX*, *CDKN1A*, *CDH1*, *CDH2*, *VIM*, *SLC7A11*, *GPX4*, *ACSL4*, *HIF1A*, *EPAS1*, and *TP53*. Pre-Developed TaqMan^TM^ Assay Reagents (Applied Biosystems) were used for *ACTB* as an internal control. PCR reactions were carried out with a 7500 Real-Time PCR System (Applied Biosystems) under standard conditions. Relative gene expression levels were calculated by using *ACTB* expression as the denominator for each cell line.

### Statistical analysis

Statistical tests were performed using SPSS Statistics version 17.0 (IBM). For comparing paired groups, data were analyzed with Student’s *t* test. For comparing more than three groups, data were analyzed with a one-way ANOVA test, and Dunnett’s test or the Tukey-Kramer HSD test was used for post-hoc group comparisons. *p* ≤ 0.05 was considered statistically significant.

## Results

### Cytotoxic activity of LF on various cell lines under normoxic or hypoxic conditions

**FIGURE 1:**
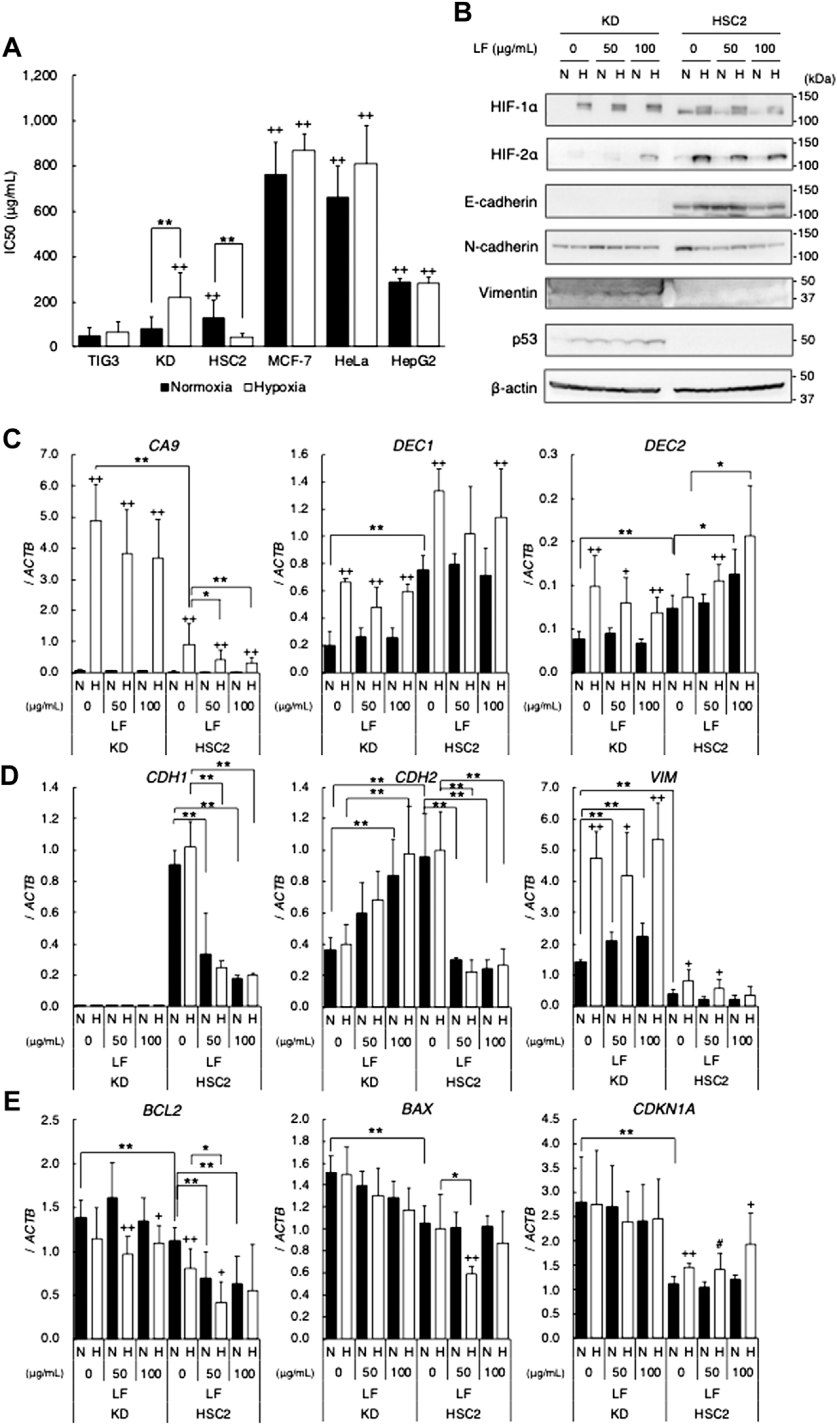
Cytotoxic activity of lactoferrin in various cell lines under normoxic (21% O_2_) or hypoxic (1% O_2_) conditions. **(A)** Cytotoxic activity evaluated as IC_50_ values with the MTT assay. Statistical significance is presented as + +: *p* < 0.01 (vs. TIG-3), **: *p* < 0.01 (indicated paired samples). **(B)** Effects of LF treatment on HIFs (HIF-1α and HIF-2α), EMT markers (E-cadherin, N-cadherin, and vimentin), and p53 proteins in KD and HSC2 under normoxic (N) and hypoxic (H) conditions for 24 h evaluated by using immunoblot analyses. Expression level of β-actin was used as a loading control. A representative result is shown (*n* = 3). **(C)** Effects of LF treatment on expression of HIF-1 target genes, *CA9*, *DEC1*, and *DEC2*. **(D)** EMT marker genes, *CDH1*, *CDH2*, and *VIM*. **(E)** Apoptosis and cell cycle related genes, *BCL2*, *BAX*, and *CDKN1A*, in KD and HSC2 under normoxic (N) and hypoxic (H) conditions for 24 h evaluated by using quantitative RT-PCR. Relative gene expression level was calculated by using *ACTB* expression as the denominator for each cell line (*n* = 3). For all quantitative values, the average and SD are shown. Statistical significance is represented as +: *p* < 0.05 and ++: *p* < 0.01 (N vs H); and #: *p* = 0.05, *: *p* < 0.05, and **: *p* < 0.01 (indicated paired samples).

In order to clarify whether LF exerts cytotoxic activity on various cell lines under normoxic and hypoxic conditions, IC_50_ values were evaluated with the MTT assay. LF showed cytotoxic activity on tested cell lines with a range of sensitivities: TIG3 and KD were relatively sensitive, MCF-7 and HeLa were resistant, and HSC2 and HepG2 were intermediate under normoxic conditions (Figure 1A). Hypoxia decreased sensitivity to LF in KD but increased that in HSC2, whereas sensitivity in other cell types was not significantly changed. Immunoblot analysis revealed that LF treatment increased hypoxic HIF-1α and -2α proteins in KD but attenuated that in HSC2 cells in a dose-dependent manner (Figure 1B). LF treatment also decreased expression of HIF-1 target gene *CA9* and increased expression of *DEC2* in hypoxic HSC2 but not in KD (Figure 1C). The other target gene, *DEC1*, did not show any trend with LF treatment in either cell line. Because it is generally known that hypoxia regulates cell differentiation, especially epithelial-mesenchymal transition (EMT) in cancer cells, effects of LF on expression of EMT markers in KD and HSC2 cells were also evaluated. Immunoblot analysis revealed that epithelial marker E-cadherin was expressed in HSC2 but not in KD, mesenchymal marker vimentin was expressed in KD but not in HSC2, and N-cadherin was expressed in both cell types (Figure 1B). Furthermore, LF treatment decreased expression of E-cadherin and N-cadherin in HSC2, but increased expression of N-cadherin and vimentin in KD. Likewise, LF treatment decreased mRNA levels of *CDH1* (E-cadherin) and *CDH2* (N-cadherin) in HSC2 but increased those of *CDH2* and *VIM* (vimentin) in KD (Figure 1D). Furthermore, expression of apoptosis and cell cycle related genes was evaluated in both cell lines; *BCL2*, *BAX*, and *CDKN1A* in KD had higher levels of expression than those in HSC2. LF treatment, especially in HSC2, decreased expression of *BCL2* and seemed to enhance hypoxic induction of *CDKN1A* (Figure 1E). Protein levels of tumor suppressor p53, which regulates transcription of target genes including *BAX* and *CDKN1A*, were increased with LF treatments and further enhanced under hypoxic conditions in KD, but p53 was not detected in HSC2 (Figure 1B).

### p53 status modifies cytotoxic activity of lactoferrin

**FIGURE 2:**
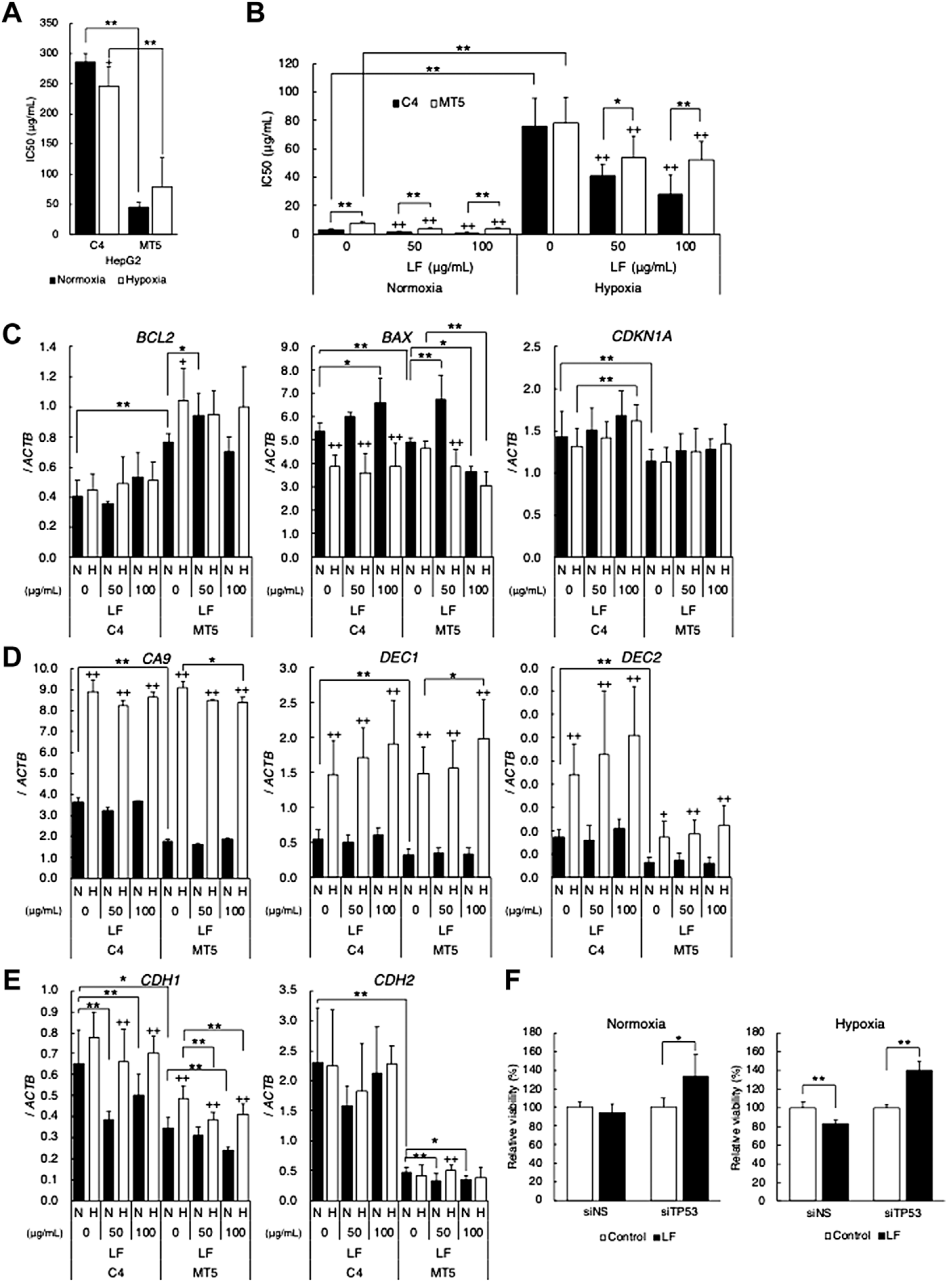
p53 status modifies cytotoxic activity of lactoferrin. Cytotoxic activity of **(A)** lactoferrin (LF) or **(B)** bleomycin on HepG2 transfected with pCMX empty plasmid vector (C4) or pCMX-p53-R248W (MT5) under normoxic (21% O_2_) and hypoxic (1% O_2_) conditions evaluated as IC_50_ values by using the MTT assay. Statistical significance is presented as ++: *p* < 0.01 (vs. LF 0 μg/ml of each clone), *: *p* < 0.05, and **: *p* < 0.01 (indicated paired samples). **(C)** Effects of LF treatment on expression of apoptosis and cell cycle related genes. **(D)** HIF-1 target genes. **(E)** EMT marker genes in C4 and MT5 under normoxic (N) and hypoxic (H) conditions for 24 h evaluated by using quantitative RT-PCR. Relative gene expression level was calculated by using *ACTB* expression as the denominator for each cell line (*n* = 3). For all quantitative values, the average and SD are shown. Statistical significance is represented as +: *p* < 0.05, and ++: *p* < 0.01 (N vs H), *: *p* < 0.05, and **: *p* < 0.01 (indicated paired samples). **(F)** Cytotoxic activity of lactoferrin (LF) on HepG2 transfected with control siRNA (siNS) or *TP53* specific siRNA (siTP53) under normoxic (left) and hypoxic (right) conditions evaluated by using the MTT assay. Relative viability was calculated as percentage (%) of control (without LF treatment) viability. Statistical significance is presented as *: *p* < 0.05, and **: *p* < 0.01 (indicated paired samples).

Because tumor suppressor p53 is one of the most important regulators of apoptosis and the cell cycle, HepG2 expressing mutant-type p53 was employed to clarify the role of p53-signal in cytotoxic activity of LF. MTT assay revealed that forced expression of mutant-type p53 in HepG2 (MT5 clone) increased sensitivity to LF (decreased IC_50_ value), strikingly in comparison with empty-vector transfected HepG2 (C4 clone) (Figure 2A). Since p53 status is also well known to affect sensitivity to DNA-damage inducing anticancer drugs, IC_50_ values of Bleomycin in LF-treated HepG2 were evaluated. Sensitivity to Bleomycin in MT5 was significantly higher than in C4, and hypoxia drastically decreased sensitivity in both clones (Figure 2B). LF treatment significantly sensitized both clones to Bleomycin, regardless of oxygen condition, but LF worked more effectively in C4 than in MT5. Level of *BCL2* expression was higher and that of *CDKN1A* was lower in MT5 than in C4, and LF treatment further increased expression of *CDKN1A* in C4 (Figure 2C). Level of *BAX* expression was decreased in hypoxic C4, and LF treatment increased it in normoxic C4 and decreased it in hypoxic MT5. Expression of *CA9*, *DEC1*, and *DEC2* increased in both clones under hypoxic conditions, but was not changed with LF treatment (Figure 2D). Expression of *DEC2* was significantly lower in MT5 than in C4, suggesting *DEC2* regulation by mutant-type p53. Expression of *CDH1* and *CDH2* seemed to decrease with LF treatment, and expression of *CDH2* was significantly lower in MT5, the same as *DEC2* (Figure 2E). Because p53 signal seemed to play a role in response to LF, knock-down experiments using siRNA were performed in HepG2. Knock-down of *TP53* (p53 gene) increased cell viability of LF-treated HepG2 under normoxic and hypoxic conditions (Figure 2F).

### Possible involvement of ferroptosis in cytotoxic activity of lactoferrin under hypoxic conditions

**FIGURE 3:**
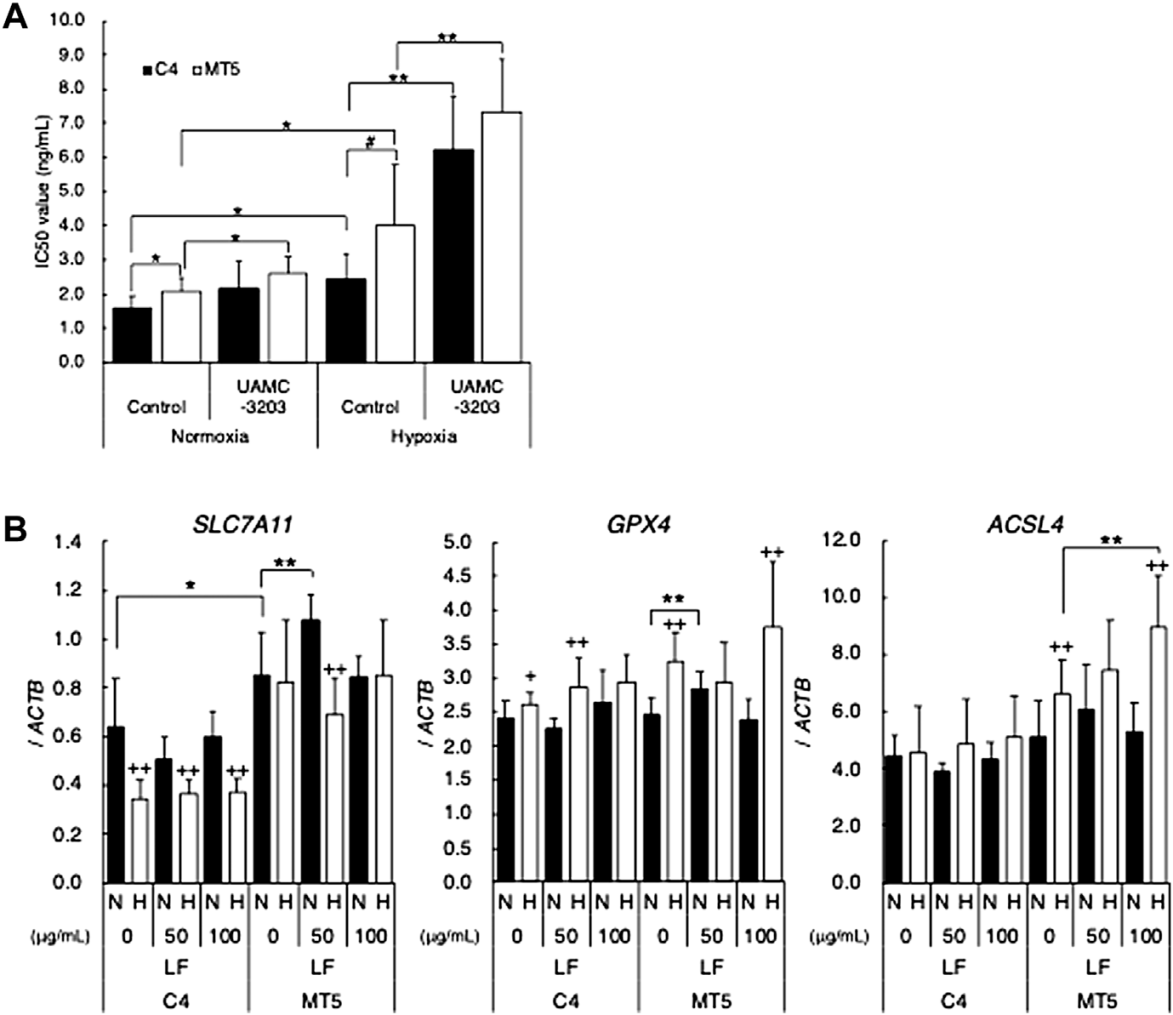
Possible involvement of ferroptosis in cytotoxic activity of lactoferrin under hypoxic conditions. **(A)** Cytotoxic activity of Erastin on HepG2 (C4) or (MT5) under normoxic (21% O_2_) and hypoxic (1% O_2_) conditions without or with ferroptosis inhibitor, UAMC-3203, were evaluated as IC_50_ values by using the MTT assay. Statistical significance presented as *: *p* < 0.05, and **: *p* < 0.01 (indicated paired samples). **(B)** Effects of LF treatment on feroptosis related genes, *SLC7A11*, *GPX4*, and *ACSL4*, in C4 and MT5 under normoxic (N) and hypoxic (H) conditions for 24 h evaluated by using quantitative RT-PCR. Relative gene expression level was calculated by using *ACTB* expression as the denominator for each cell line (*n* = 3). For all quantitative values, the average and SD are shown. Statistical significance is represented as +: *p* < 0.05, and ++: *p* < 0.01 (N vs H), *: *p* < 0.05, **: *p* < 0.01, and #: *p* = 0.05 (indicated paired samples).

In order to clarify the mechanism of cytotoxic activity of lactoferrin through p53 signal, possible involvement of ferroptosis was determined. At first, sensitivity of Erastin, which is known as a ferroptosis-inducing reagent, was evaluated in C4 and MT5, and MTT assay revealed that Erastin-induced ferroptosis seemed to occur in both C4 and MT5 (Figure 3A). IC_50_ values were higher in MT5 under normoxic and hypoxic conditions than in C4, and hypoxia significantly decreased sensitivity to Erastin in both clones. A ferroptosis inhibitor, UAMC-3203, drastically decreased the Erastin sensitivity in both clones under hypoxic conditions, suggesting hypoxia induced ferroptosis in those cells. Expression of the ferroptosis-regulating genes *SLC7A11*, *GPX4*, and *ACSL4* was further evaluated in LF-treated C4 and MT5 under normoxic and hypoxic conditions (Figure 3B). Expression of *SLC7A11* was higher in MT5 than in C4 and decreased in hypoxic C4 but not in MT5. Expression of *GPX4* was slightly increased in hypoxic C4 and MT5 clones. Interestingly, expression of *ACSL4* was increased in hypoxia and LF-treated MT5 but not in C4, suggesting an increase of ferroptosis just in LF-treated cells with mutant-type p53.

## Discussion

In this study, inhibitory effects of LF on cell proliferation were observed in cell lines with a wide variety of sensitivities to LF. Firstly, regardless of whether cells were normal or malignant, LF inhibited cell proliferation of normal fibroblast (TIG-3 and KD), squamous cell carcinoma (HSC2), and hepatoma (HepG2) cells. LF also inhibited proliferation of MCF-7 and HeLa cells with significantly higher IC_50_ value than other cells, suggesting that cells derived from female-specific organs may have distinct mechanisms to resist cytotoxicity of LF. Interestingly, LF treatments oppositely affected KD and HSC2 under hypoxic conditions: Hypoxia decreased sensitivity (increased IC_50_ value) to LF in KD but increased sensitivity (decreased IC_50_ value) in HSC2. In order to clarify the molecular mechanisms of differential effects of LF on those cells, cellular characteristics were compared between those 2 cell types. At first, the effect of LF on levels of HIF-1α and -2α proteins, master regulators of the hypoxic transcriptome, were determined. Hypoxia-stabilized HIF-1α and -2α proteins in KD were strikingly found to be increased with LF treatments, but those in HSC2 were decreased. On the other hand, expression levels of *HIF1A* (HIF-1α gene) and *EPAS1* (HIF-2α gene) did not increase in LF-treated or hypoxic KD and were not altered in HSC2, suggesting that LF affected the translational or post-translational process to regulate these proteins (Figure 1B, Supplemental Figure). It was also interestingly shown that LF affected levels of HIF-1α and HIF-2α proteins similarly, suggesting a common mechanism such as mTOR signal or PHD-pVHL axis (Albanese et al., 2021). Expressions of *CA9*, which is one of the most HIF-dependent target genes, in HSC2 decreased with LF treatment under hypoxic conditions concomitant with HIF-α proteins. However, its expression also slightly decreased in KD, in which HIF-1α protein level was increased with LF treatment. Although *DEC1* did not show any trend of gene expression, *DEC2* interestingly showed an opposite trend between KD and HSC2: LF treatment decreased *DEC2* in KD but increased that in HSC2, suggesting a possible relationship between LF-modified *DEC2* expression and HIF-1α protein level. Because a limited number of genes was analyzed in this study, further transcriptome analyses would be interesting if it could lead to identification of the functional genes involved in the response to LF.

Secondly, histological differences between KD and HSC2 were considered, since KD is derived from mesenchymal tissue and HSC2 from epithelial tissue. Epithelial marker E-cadherin was detected in HSC2 but not in KD, and mesenchymal marker vimentin was detected in KD but not in HSC2. N-cadherin was detected in both cell types. Hypoxia seemed to increase E-cadherin protein in HSC2 and LF treatment slightly decreased it. Hypoxia seemed not to modify vimentin protein level in KD, but LF treatment increased it. N-cadherin decreased in hypoxic HSC2 but not in KD, and increased in LF-treated KD but decreased in HSC2. RT-PCR analyses clearly demonstrated that expression of those proteins was regulated at the mRNA level: expression of *CDH1* and *CDH2* in HSC2 decreased with LF treatment whereas *CDH2* and *VIM* in KD increased with LF treatment. These results indicate that LF treatment enhances mesenchymal phenotype in KD but causes a loss of histological features in HSC2, suggesting that the signal relating to mesenchymal features, especially N-cadherin, might be associated with response to LF. A previous study demonstrated that bLF treatment increases expression of E-cadherin but decreases that of vimentin in an oral squamous cell carcinoma line, HOC313, in which the EMT signal was induced, resulting in an inhibition of EMT (Chea et al., 2018a). Another study also reported that bLF treatment decreased expression of snail and vimentin but increased that of cadherin in a glioblastoma cell line (Cutone et al., 2020). Although those reports did not support our findings, the molecular mechanisms of cell-type-specific differential functions of LF need to be further investigated as our study suggested.

Finally, one of the well-known differences between benign (KD) and malignant (HSC2) cell lines was clear: head and neck cancers, including oral squamous cell carcinoma, are generally recognized as having the highest rate of p53 mutation (Olivier et al., 2010), and KD has wild-type p53 but HSC2 has mutant-type p53 (NM_001126113.3:c.672 + 1G > A) (JCRB Cell Bank, 1997). In this study, p53 protein was actually detected in KD but not in HSC2. p53 is one of the most important tumor suppressor proteins regulating apoptosis and the cell cycle (Aubrey et al., 2018; Pitolli et al., 2019). LF treatment decreased *BCL2* expression and slightly increased *CDKN1A* in HSC2, suggesting inhibition of anti-apoptotic signal and induction of cell cycle arrest in HSC2 under hypoxic conditions. A previous report similarly demonstrated that bLF activates p53 and induces p21 in cancer cells, resulting in induction of G1/S arrest (Chea et al., 2018b). Because LF seemed to regulate apoptosis and the cell cycle in cells with mutant-type p53, we employed HepG2 expressing mutant-type p53, as previously reported (Fukazawa et al., 2019). HepG2 originally expresses wild-type p53. Therefore, transfected mutant-type p53 works as a dominant-negative form in HepG2 (MT5), resulting in a modified p53 signal (Fukazawa et al., 2019). MTT assay strikingly demonstrated that cells expressing mutant-type p53 (MT5) were significantly more sensitive to LF than control HepG2 (C4), suggesting an important role of the p53 signal in LF sensitivity. Level of *BCL2* expression was higher, and levels of *BAX* and *CDKN1A* were lower in MT5 than in C4, resulting in phenotypes with anti-apoptotic and progressive cell cycle (Labi et al., 2008; Borrero and El-Deiry, 2021; Chota et al., 2021; Engeland, 2022). In fact, MT5 is more resistant than C4 to the anti-cancer drug Bleomycin. Hypoxia drastically decreased sensitivity to Bleomycin, but LF treatment sensitized both clones, suggesting potential application as a sensitizer or enhancer to anti-cancer therapies. The hypoxia-inducible gene *DEC2* and the histological marker gene *CDH2* were expressed at lower levels in MT5 under both normoxic and hypoxic conditions, suggesting that DEC2 and N-cadherin might contribute to differences between C4 and MT5. LF treatment further decreased the level of *CDH2* expression just in MT5. Taken together, mutant-type p53-modified expression of target genes, especially *DEC2* and *CDH2*, might contribute to differential response to LF treatment. Knock-down of *TP53* interestingly reduced sensitivity to LF in HepG2 under normoxic and hypoxic conditions, although inhibition of p53 signal was expected to sensitize to LF. This might suggest that p53 signal itself is a target of LF cytotoxic activity, and loss of p53 signal would imply loss of the target of LF, resulting in decreased sensitivity or an alteration of determinants. In this study, LF sensitivity decreased in hypoxic KD, in which levels of p53 protein increased, however it increased in hypoxic HSC2, in which p53 protein was not detected. Namely, mutant-type p53 in MT5 might modify p53 signal to cause a gain of some functions that are targets of LF, or other sensitivity determinants, possibly HIFs, might dominate in HSC2. Previous reports demonstrated that LF specifically upregulates *TP53* gene expression in HeLa cells through the activation of NF-κB, resulting in activation of p53 downstream signal (Oh et al., 2004), but bLF also induces the phosphorylation of p53 and induces p21 in cancer cells (Chea et al., 2018a). Those reports clearly indicate p53 as one of the targets of LF, although effects of LF on mutant-type p53 or p53 status had not yet been determined.

To further clarify detailed mechanisms of the cytotoxic activity of LF, we focused on ferroptosis, which is a newly identified mechanism of cell death in the p53 downstream signal. The p53 signal pathway has recently been found to sensitize cells to ferroptosis by inhibiting SLC7A11 (Jiang et al., 2015; Tarangelo et al., 2018; Liu et al., 2020). The ACSL4/LPCAT3/LOX signal axis is also known as an intracellular pathway that promotes lipid peroxidation in ferroptosis (Yuan et al., 2016; Tang et al., 2021). Erastin was used to check the ferroptosis activity in cell lines with the different p53 status. Erastin is a ferroptosis inducer that inhibits the cysteine transporter system, resulting in inhibition of antioxidation by the glutathione peroxidase (GPX) family (Dixon et al., 2014). Although Erastin inhibited cell viability of both HepG2 C4 and MT5, MT5 was significantly more resistant to Erastin than C4. Hypoxia further reduced sensitivity to Erastin in both clones. A ferroptosis inhibitor, UAMC-3203, drastically decreased sensitivity to Erastin in both clones under hypoxic conditions, suggesting that hypoxia induces ferroptosis in those cells. In fact, ferroptosis inhibiting *SLC7A11* decreased in hypoxic C4 and promoting *ACSL4* increased in hypoxic MT5, suggesting that mechanisms of hypoxia-induced ferroptosis are different in those clones. Importantly, LF treatment increased *ACSL4* only in hypoxic MT5, suggesting the existence of LF-induced ferroptosis in cells expressing mutant-type p53.

In conclusion, a wide variety of sensitivities to cytotoxic activity of LF was observed in various cell lines, and hypoxia was found to regulate LF sensitivity differently among different cell lines, possibly through the p53 signaling pathway including DEC2 and N-cadherin. LF was further suggested to regulate ferroptosis through expression of *ACSL4*, depending on p53 status. Although further investigation including comparative analyses using multiple cell lines, comprehensive analyses and animal experiments is necessary to reach a definitive conclusion, this study provides novel insight into the clinical application of lactoferrin.

## Data Availability

The original contributions presented in the study are included in the article/Supplementary Material, further inquiries can be directed to the corresponding author.
