# Supplementary material for: p53 status modifies cytotoxic activity of lactoferrin under hypoxic conditions
Source: Front Pharmacol. 2022 Sep 19;13:988335. doi: 10.3389/fphar.2022.988335 (PMC9527284; doi:10.3389/fphar.2022.988335)
Supplement: Supplementary file 1 [file DataSheet1.docx]

**Supplementary Material**

**Table|** Primer and probe sets for quantitative RT-PCR

*CA9*-F: 5’-CCTTTGCCAGAGTTGACGAG-3’

*CA9*-R: 5’-GCAACTGCTCATAGGCACTG-3’

*CA9*-probe: UPL #25 (Roche)

*DEC1*-F: 5’-GACTGGAGCACGGAGACCT-3’

*DEC1*-R: 5’-GGTGCGGCAATTTGTAGG-3’

*DEC1*-probe: UPL #56 (Roche)

*DEC2*-F: 5’-CTACTGCGTGCCCGTCAT-3’

*DEC2*-R: 5’-CGGTGTCCGTGTCGTTCT-3’

*DEC2*-probe: UPL #26 (Roche)

*BCL2*-F: 5’-AGTACCTGAACCGGCACCT-3’

*BCL2*-R: 5’-GCCGTACAGTTCCACAAAGG-3’

*BCL2*-probe: UPL #75 (Roche)

*BAX*-F: 5’-CCATCATGGGCTGGACAT-3’

*BAX*-R: 5’-CACTCCCGCCACAAAGAT-3’

*BAX*-probe: UPL #69 (Roche)

*CDKN1A*_F: 5’-TCACTGTCTTGTACCCTTGTGC-3’

*CDKN1A*_R: 5’-GGCGTTTGGAGTGGTAGAAA-3’

*CDKN1A*_Probe: UPL #32 (Roche)

*CDH1*_F: 5’-GGCCAGGAAATCACATCCTA -3’

*CDH1*_R: 5’-GGCAGTGTCTCTCCAAATCC-3’

*CDH1*_Probe: UPL #36 (Roche)

*CDH2*_F: 5’-CTCCATGTGCCGGATAGC-3’

*CDH2*_R: 5’-CGATTTCACCAGAAGCCTCTAC-3’

*CDH2*_Probe: UPL #74 (Roche)

*VIM*_F: 5’-GTTTCCCCTAAACCGCTAGG-3’

*VIM*_R: 5’-AGCGAGAGTGGCAGAGGA-3’

*VIM*_Probe: UPL #56 (Roche)

*SLC7A11*_F: 5’-CCATGAACGGTGGTGTGTT-3’

*SLC7A11*_R: 5’-GACCCTCTCGAGACGCAAC-3’

*SLC7A11*_Probe: UPL #80 (Roche)

*GPX4*_F: 5’-ACTTCACCAAGTTCCTCATCG-3’

*GPX4*_R: 5’-GCCACACACTTGTGGAGCTA-3’

*GPX4*_Probe: UPL #43 (Roche)

*ACSL4*_F: 5’-TGCACCTCTTTGCAATCTGT-3’

*ACSL4*_R: 5’-GACAGCATCATGCGGACAT-3’

*ACSL4*_Probe: UPL #3 (Roche)

*HIF1A*_F: 5’-GAACCTGATGCTTTAACTTTGCT-3’

*HIF1A*_R: 5’-TGCTGGTCATCAGTTTCTGTG-3’

*HIF1A*_Probe: UPL #28 (Roche)

*EPAS1*_F: 5’-GACATGAAGTTCACCTACTGTGATG-3’

*EPAS1*_R: 5’-GCGCATGGTAGAATTCATAGG-3’

*EPAS1*_Probe: UPL #17 (Roche)

*TP53*_F: 5’-CCCCAGCCAAAGAAGAAAC-3’

*TP53*_R: 5’-AACATCTCGAAGCGCTCAC-3’

*TP53*_Probe: UPL #58 (Roche)

**Figure|** Effects of LF treatment on expression of Key transcription factor genes, *HIF1A* (HIF-1α gene), *EPAS1* (HIF-2α gene), and *TP53* (p53 gene) in KD and HSC2 under normoxic (N) and hypoxic (H) conditions for 24 hours evaluated by using quantitative RT-PCR. Relative gene expression level was calculated by using *ACTB* expression as the denominator for each cell line (n = 3). For all quantitative values, the average and SD are shown. Statistical significance is represented as +: *P* < 0.05 and ++: *P* < 0.01 (N vs H); *: *P* < 0.05, and **: *P* < 0.01 (indicated paired samples).

**Raw data|** Scanned images for Figure 1B
